# Supplementary material for: MetaRibo-Seq measures translation in microbiomes
Source: Nat Commun. 2020 Jun 29;11:3268. doi: 10.1038/s41467-020-17081-z (PMC7324362; doi:10.1038/s41467-020-17081-z)
Supplement: Supplementary file 10 — Supplementary Data 7 [file 41467_2020_17081_MOESM10_ESM.zip › File2/Confidence_High_Taxonomy/169902_out.krona.html]

Javascript must be enabled to view this page.

members
magnitude
magnitudeUnassigned
count
unassigned
taxon
rank

169902\_out

5

2
superkingdom
5

976
phylum
5

class
200643
5

order
171549
5

815
family
5

5
816
genus

2

SRS1055067\_contig\_number\_16074SRS144603\_contig\_number\_contig-100\_18940.18941
2292941
species

2
species
204516

SRS019161\_contig\_number\_15118SRS893383\_contig\_number\_26441

1

SRS1041037\_contig\_number\_8316
species
2292944
